# Efficacy and safety of live combined *Bacillus subtilis* and *Enterococcus faecium* in patients with *Helicobacter pylori* infection: A meta-analysis of randomized controlled trials

**DOI:** 10.1016/j.clinsp.2026.101139

**Published:** 2026-09-10

**Authors:** Chenghai Yang, Bingyun Lu, Cangui Zhang, Anjiang Wang, Ye Chen

**Affiliations:** Integrative Microecology Clinical Center, Shenzhen Key Laboratory of Gastrointestinal Microbiota and Disease, Shenzhen Clinical Research Center for Digestive Disease, Shenzhen Hospital, Southern Medical University, Shenzhen, China

**Keywords:** *Helicobacter Pylori* infection, Live combined *Bacillus Subtilis* and *Enterococcus Faecium*, Efficacy, Safety, Immunity and inflammation

## Abstract

**Objective:**

Live Combined *Bacillus subtilis* and *Enterococcus faecium* (LCBE) regulates gut microbiota and gastrointestinal inflammation. This meta-analysis aimed to compare the efficacy and safety of interventions with or without LCBE in patients with *Helicobacter pylori* (*H. pylori*) infection.

**Methods:**

Web of Science, PubMed, Cochrane Library, China National Knowledge Infrastructure, Wan Fang and China Science and Technology Journal Database were searched until February 2025. Patients with *H. pylori* infection receiving interventions with or without LCBE were assigned to the experimental and control groups, respectively. This meta-analysis was retrospectively registered in PROSPERO (CRD420261430266).

**Results:**

A total of 26 randomized controlled trials containing 3,536 patients with *H. pylori* infection were included. *H. pylori* eradication rate in the experimental group was 2.403-times of that in the control group. The total adverse event rate in the experimental group was 0.329-times of that in the control group. Diarrhea rate, nausea and vomiting rate, abdominal distension rate, poor appetite rate and constipation rate were lower in the experimental group than those of the control group. With regard to non-gastrointestinal adverse events, only taste disorder rate was lower in the experimental group than that of the control group. The expression levels of immunoglobulins A and G were increased, while those of tumor necrosis factor-alpha and interleukin-6 were decreased in the experimental group compared with those noted in the control group.

**Conclusion:**

LCBE-containing interventions are associated with higher eradication rates, fewer adverse events, and favorable changes in immune and inflammatory markers in patients with *H. pylori* infection.

## Highlights

• A meta-analysis with 26 randomized controlled trials containing 3,536 patients.
• LCBE supplementation increases *Helicobacter pylori* eradication rate.
• LCBE supplementation reduces total and gastrointestinal adverse events.
• LCBE supplementation increases Immunoglobulin (Ig)A and IgG levels.
• LCBE supplementation reduces tumor necrosis factor-α and interleukin-6 levels.

## Introduction

*Helicobacter pylori* (*H. pylori*) infection affects ⁓50% of the global population and is the leading cause of chronic gastritis, peptic ulcers and gastric cancer.^1^ Current recommended therapies are antibiotic-based triple or quadruple regimens.^2^, ^3^, ^4^ However, the occurrence of antibiotic resistance impairs the efficacy of these therapies, which leads to declining eradication rates.^5^^,^^6^ In addition, adverse effects associated with antibiotic use, including gastrointestinal disturbances and microbial dysbiosis, increase safety concerns.^7^^,^^8^ Therefore, exploring the development of potential adjunctive regimens to improve efficacy while minimizing adverse events is crucial in enhancing the management of patients with *H. pylori* infection.

Probiotics are beneficial live microorganisms that are commonly used as an adjunctive regimen in antibiotics for the treatment of *H. pylori* infection.^9^ Probiotics can enhance *H. pylori* eradication by producing antibacterial substances, reducing adhesion, attenuating inflammation and enhancing immune responses.^9^ Furthermore hand, probiotics can also regulate the gut microbiota to alleviate adverse events caused by antibiotics in patients with *H. pylori* infection.^10^

Live Combined *Bacillus subtilis* and *Enterococcus faecium* (LCBE), a probiotic formulation, possesses the ability to regulate gut microbiota and gastrointestinal inflammation.^11^^,^^12^ Certain Randomized Controlled Trials (RCTs) have investigated the efficacy and safety of LCBE-containing regimens in patients with *H. pylori* infection.^13^, ^14^, ^15^, ^16^, ^17^, ^18^, ^19^, ^20^, ^21^, ^22^, ^23^, ^24^, ^25^, ^26^, ^27^, ^28^, ^29^, ^30^, ^31^, ^32^, ^33^, ^34^, ^35^, ^36^, ^37^, ^38^ Nevertheless, the findings of these RCTs are inconsistent. In addition, the sample sizes of certain RCTs were relatively small, which impaired the statistical power. Therefore, to provide a holistic representation of the efficacy and safety of LCBE in patients with *H. pylori* infection, a meta-analysis is required.

This meta-analysis aimed to compare the efficacy and safety of interventions with or without LCBE in patients with *H. pylori* infection.

## Materials and methods

### Registration

This meta-analysis was conducted in accordance with the Preferred Reporting Items for Systematic Reviews and Meta-Analyses (PRISMA) guidelines.^39^ The protocol was retrospectively registered in PROSPERO (CRD420261430266).

### Literature strategy

A comprehensive search was conducted across multiple databases, including Web of Science, PubMed, Cochrane Library, China National Knowledge Infrastructure, Wan Fang, and China Science and Technology Journal Database (VIP). The search utilized keywords such as “Bacillus Subtilis and Enterococcus Faecium”, “Live Combined Bacillus Subtilis and Enterococcus Faecium”, “Medilac-Vita”, “Medilac-S”, “Probiotics”, “Helicobacter pylori infection”, “H. pylori infection”, and “HP infection”. The search was limited to studies published prior to February 2025. To ensure comprehensiveness, the references of the selected studies were manually reviewed to identify potentially relevant studies. The detailed search strategies for all databases are presented in Supplementary Table 1.

**Table 1:** Overview of included studies.

**Study ID**	**Demographics^a^**		**Sample size**		**Disease type**	**Intervention**		**LCBE regimen**
**First author (publication year)**	**Experimental group**	**Control group**	**Experimental group**	**Control group**		**Experimental group**	**Control group**	
Liu ZP (2009)^13^	Age (all): PU: 35.6±11.5; CG: 44.3±9.3		100	100	PU or CG	LCBE + Triple therapy	Triple therapy	500 mg, tid; After meal; 2-weeks
	Sex (all): PU: 54/26; CG: 55/65							
Zhang QZ (2012)^14^	Age: 46.6±10.7	Age: 49.7±12.3	60	66	PU or CG	LCBE + Triple therapy	Triple therapy	500 mg, tid; After meal; 2-weeks
	Sex: 33/27	Sex: 35/31						
Hu LY (2013)^15^	Age (all): 36.5±7.2		118	112	Not report	LCBE + Triple therapy	Triple therapy	500 mg, tid; After meal; 2-weeks
	Sex (all): 112/118							
Duan ZY (2014)^16^	Age: 44.2±3.8	Age: 42.4±5.8	80	80	Not report	LCBE + Triple therapy	Triple therapy	500 mg, tid; After meal; 1-week
	Sex: 42/38	Sex: 39/41						
He QL (2014)^17^	Age (all): DU: 38.2±11.5; CEG: 40.4±10.4		150	150	DU or CEG	LCBE + Quadruple therapy	Quadruple therapy	500 mg, bid; Before meal; 10-days
	Sex (all): 209/91							
Jiang ZY (2014)^18^	Age (all): 36.5±7.2		50	46	PU	LCBE + Triple therapy	Triple therapy	500 mg, tid; After meal; 10-days
	Sex (all): 52/44							
Du HW (2015)^19^	Age (all): 60.0‒87.0		112	112	CEG, GU, or DU	LCBE + Quadruple therapy	Quadruple therapy	500 mg, tid; 2-weeks
	Sex (all): 128/96							
Zhang JF (2015)^20^	Age: 40.2±3.2	Age: 40.1±3.0	102	102	PU	LCBE + Quadruple therapy	Quadruple therapy	500 mg, bid; Before meal; 10-days
	Sex: 64/38	Sex: 66/36						
Cui Y (2016)^21^	Age: 38.9±14.0	Age: 34.2±13.3	30	31	Not report	LCBE + Triple therapy	Triple therapy	500 mg, tid; 2-weeks
	Sex: 15/15	Sex: 15/16						
Oh B (2016)^22^	Age: 51.7±4.8	Age: 49.3±3.6	10	10	GU or DU	LCBE + Triple therapy	Triple therapy	bid; 2-weeks
	Sex: 7/3	Sex: 7/3						
Xu P (2016)^23^	Age: 44.0±10.0	Age: 42.0±11.0	34	36	Not report	LCBE + Quadruple therapy	Quadruple therapy	500 mg, tid; 2-weeks
	Sex: 18/16	Sex: 19/17						
Luo QT (2017)^24^	Age: 41.5±12.9	Age: 39.4±13.1	80	80	DU	LCBE + Quadruple therapy	Quadruple therapy	500 mg, tid; Before meal; 2-weeks
	Sex: 52/28	Sex: 50/30						
Wang Y (2017)^25^	Age: 45.5±6.5	Age: 46.0±5.6	135	132	FD	LCBE + Triple therapy	Triple therapy	500 mg, tid; 2-weeks
	Sex: 69/66	Sex: 65/67						
Zhou QF (2017)^26^	Age: 70.5±7.1	Age: 72.5±7.4	46	46	PU	LCBE + Quadruple therapy	Quadruple therapy	500 mg, tid; After meal; 2-weeks
	Sex: 24/22	Sex: 27/19						
Chen X (2018)^27^	Age: 67.3±7.5	Age: 66.6±7.2	50	50	Not report	LCBE + Quadruple therapy	Quadruple therapy	500 mg, tid; 2-weeks
	Sex: 34/16	Sex: 31/19						
Jin L (2018)^28^	Age: 38.6±10.7	Age: 36.4±13.3	60	60	CG or CEG	LCBE + Quadruple therapy	Quadruple therapy	500 mg, tid; Before meal; 2-weeks
	Sex: 38/22	Sex: 36/24						
Xue QY (2018)^29^	Age: 40.1±12.5	Age: 39.8±12.9	82	82	DU	LCBE + Quadruple therapy	Quadruple therapy	250 mg, bid; Before meal; 10‒14 days
	Sex: 48/34	Sex: 50/32						
Zhang DH (2018)^30^	Age: 40.1±3.6	Age: 41.5±3.1	60	60	DU	LCBE + Quadruple therapy	Quadruple therapy	500 mg, tid; 2-weeks
	Sex: 42/18	Sex: 46/14						
Chen CJ (2019)^31^	Age: 42.5±4.6	Age: 41.6±5.6	50	50	CG or CEG	LCBE + Triple therapy	Triple therapy	1000 mg, tid; Before meal; 4-weeks
	Sex: 27/23	Sex: 26/24						
Wu L (2019)^32^	Age: 45.2±11.6	Age: 45.5±8.4	20	20	DU	LCBE + Triple therapy	Triple therapy	500 mg, tid; 6-weeks
	Sex: 12/8	Sex: 13/7						
Ye MQ (2019)^33^	Age: 38.1±4.0	Age: 37.7±3.8	50	50	DU	LCBE + Quadruple therapy	Quadruple therapy	500 mg, tid; 8-weeks
	Sex: 27/23	Sex: 29/21						
Chang XL (2021)^34^	Age: 38.8±1.6	Age: 38.9±2.0	75	75	DU	LCBE + Quadruple therapy	Quadruple therapy	500 mg, tid; 2-weeks
	Sex: 47/28	Sex: 46/29						
Zhao JY (2022)^35^	Age: 8.3±2.3	Age: 9.1±3.5	60	60	Not report	LCBE + Triple therapy	Triple therapy	1 g, bid; 2-weeks
	Sex: 36/24	Sex: 40/20						
Li XM (2024)^36^	Age: 41.5±2.8	Age: 41.8±2.9	48	48	Not report	LCBE + Triple therapy	Triple therapy	500 mg, tid; 2-weeks
	Sex: 29/19	Sex: 28/20						
Li XY (2024)^37^	Age: 38.0±6.5	Age: 37.5±6.9	58	58	CG, GU, or DU	LCBE + Quadruple therapy	Quadruple therapy	500 mg, tid; 2-weeks
	Sex: 32/26	Sex: 30/28						
Lin CC (2024)^38^	Age: 32.6±3.3	Age: 32.6±3.2	50	50	Not report	LCBE + Dual therapy	Dual therapy	500 mg, bid; After meal; 2-weeks
	Sex: 33/17	Sex: 32/18						

PU, Peptic Ulcer; CG, Chronic Gastritis; LCBE, Live Combined Bacillus subtilis and Enterococcus faecium; DU, Duodenal Ulcer; CEG, Chronic Erosive Gastritis; GU, Gastric Ulcer; FD, Functional Dyspepsia.

Special statement of superscript “a”: 1) Age was described as mean ± standard deviation, or range. The unit of age was “years”. 2) Sex was described as the number of males/number of females. 3) The “all” in parentheses indicated that the data described the overall study population, and there was no separate report of experimental and control groups in the original study.

### Literature selection criteria

Eligible studies met the following inclusion criteria: 1) Studies involving patients diagnosed with *H. pylori* infection; 2) Studies comparing efficacy or safety outcomes between groups treated with LCBE plus control treatment and those receiving control treatment alone; 3) Studies published in English or Chinese. Studies were excluded if they were reviews, meta-analyses, animal studies, case reports, or non-RCTs. In addition, studies were excluded if they involved overlapping patient populations or assessments by the same authors. The quality assessment of eligible studies was conducted using the ROB2.0 tool.^40^ Literature selection and quality assessment were independently performed by two researchers. Any disagreements were resolved through discussion, and a consensus was reached.

### Data extraction

Data extraction comprised study details, such as the first author, year of publication, demographics, sample size and intervention. Outcomes related to efficacy, safety, immunity and inflammation evaluation of LCBE in treating *H. pylori* infection were systematically screened. The efficacy-related indicators included *H. pylori* eradication rate, while safety-related indicators involved adverse events and tolerability. Immunity and inflammation-related outcomes included common immune and inflammatory factor levels following treatment. Indicators reported in fewer than three studies were excluded from the meta-analysis to ensure robustness. Data extraction was conducted independently by two researchers using a standardized data collection form. Discrepancies were resolved through discussion and consensus.

### Statistical analysis

Data analyses were conducted using R software (version 4.4.2). The effect sizes were pooled using the Odds Ratio (OR) and 95% Confidence Interval (95% CI) for categorical variables, such as *H. pylori* eradication rate and adverse event incidence. Continuous outcomes, such as immune and inflammatory factor levels, were analyzed using the Standardized Mean Difference (SMD) with a 95% CI. Random-effects models were used for all analyses to account for potential clinical and methodological heterogeneity across studies. Peters’ and Egger’s tests were utilized for assessment of publication bias for categorical and continuous variables, respectively, when at least 10 studies were available. A p < 0.05 was considered to indicate potential publication bias, prompting the use of the trim-and-fill method to adjust estimates. The robustness of the results was performed by sensitivity analyses, which were completed by sequentially excluding individual studies. Subgroup analyses were performed only when at least two studies were available within each subgroup.

## Results

### Study flow

**Fig. 1:**
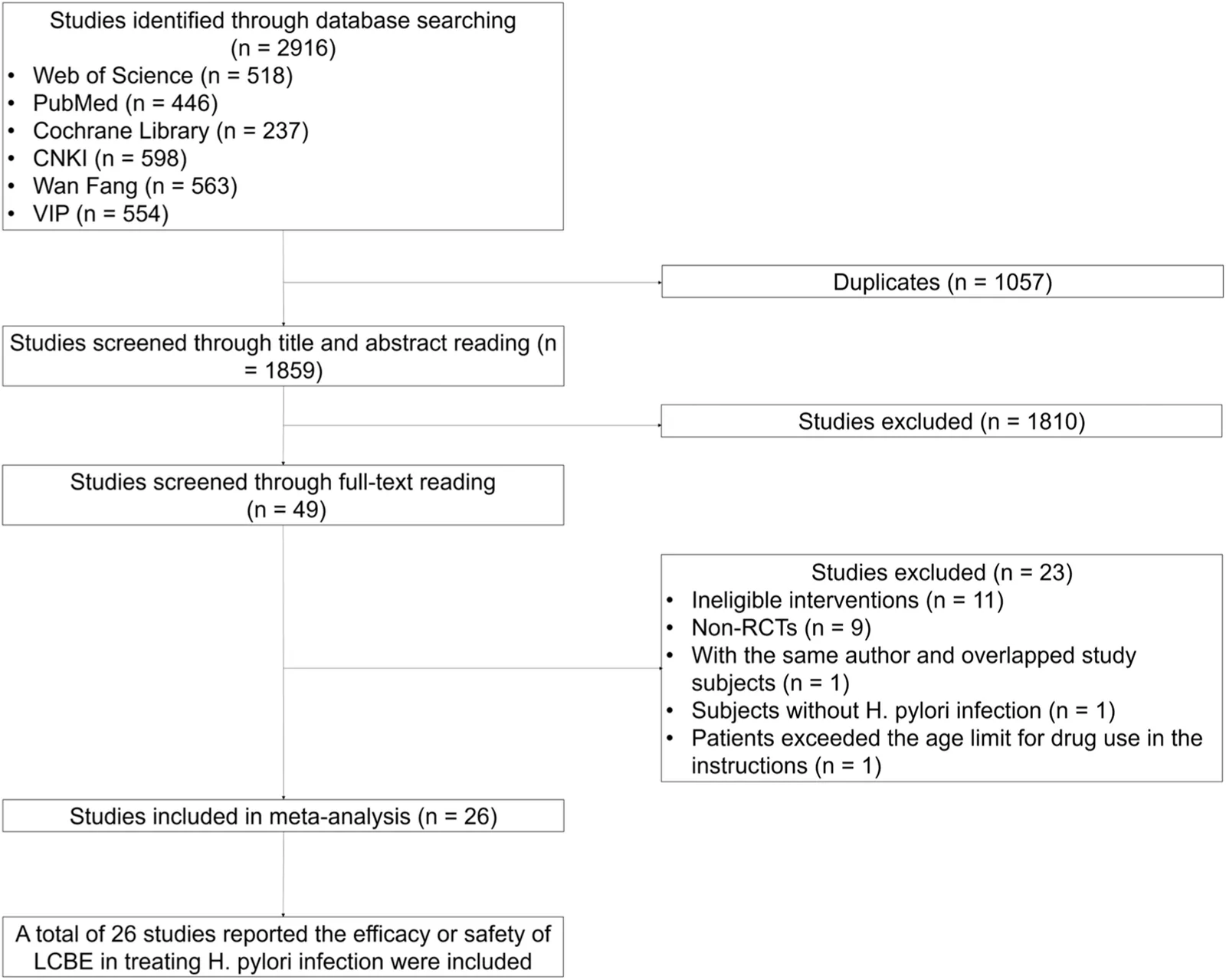
Study screen diagram.

A total of 2,916 studies were identified through database searching. Then, 1,057 duplicated studies were excluded, and 1,859 studies were screened through title and abstract reading. Subsequently, 1,810 studies were excluded. Forty-nine studies were screened through full-text reading, and 23 studies were further excluded. Twenty-six studies that reported the efficacy or safety of LCBE in treating *H. pylori* infection were included in this meta-analysis^13^, ^14^, ^15^, ^16^, ^17^, ^18^, ^19^, ^20^, ^21^, ^22^, ^23^, ^24^, ^25^, ^26^, ^27^, ^28^, ^29^, ^30^, ^31^, ^32^, ^33^, ^34^, ^35^, ^36^, ^37^, ^38^ (Fig. 1).

### Characteristics of included studies

This meta-analysis contained 3,536 patients. The age of the patients ranged from 8.3- to 87.0-years. LCBE was used in combination with triple therapy in 12 studies, with quadruple therapy in 13 studies and with dual therapy in 1 study. The dosage of LCBE was generally 500 mg, three times a day for 2-weeks in the majority of the studies. A total of 1,770 patients receiving LCBE-containing interventions were assigned to the experimental group and 1,766 patients receiving interventions without LCBE were assigned to the control group. The detailed characteristics of each included study are shown in Table 1.

### Risk of bias

**Fig. 2:**
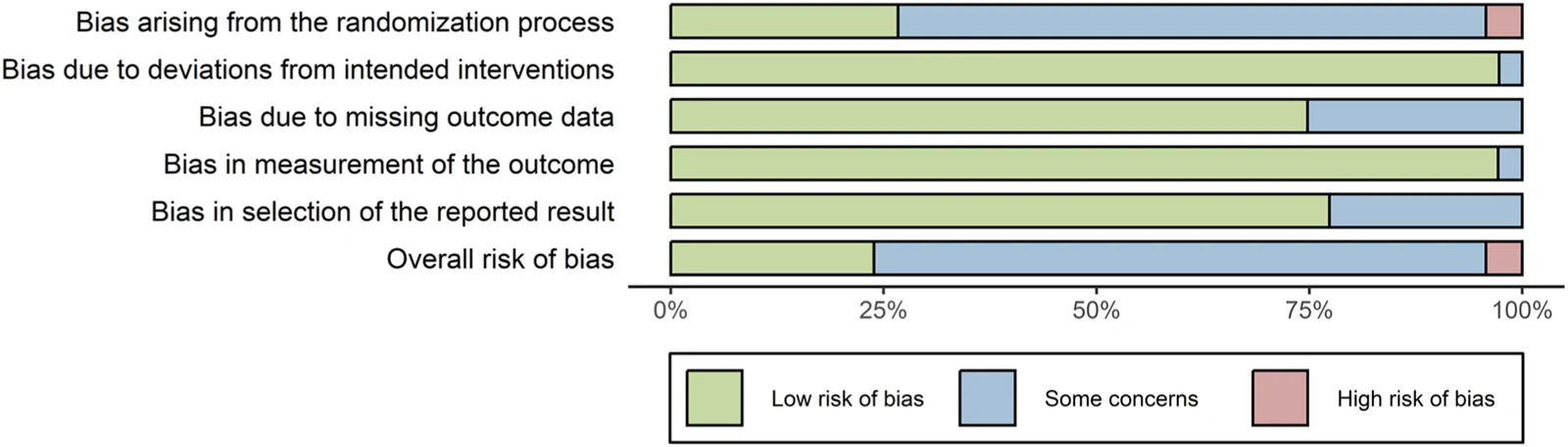
Quality assessment.

A total of 9, 25, 21, 25 and 22 studies were assessed as having low risk in bias arising from the randomization process, bias due to deviations from intended interventions, bias due to missing outcome data, bias in measurement of the outcome and bias in selection of the reported result, respectively. A total of 16, 1, 5, 1 and 4 studies were assessed as having some concerns in these 5 dimensions, respectively. One study was assessed as having high risk of bias arising from the randomization process. With regard to the overall risk of bias, 8 studies were assessed as having low risk, 17 studies were assessed as having some concerns and 1 study was assessed as having high risk (Fig. 2). The detailed information on risk of bias in each study is presented in Supplementary Figure 1.

### Efficacy

**Fig. 3:**
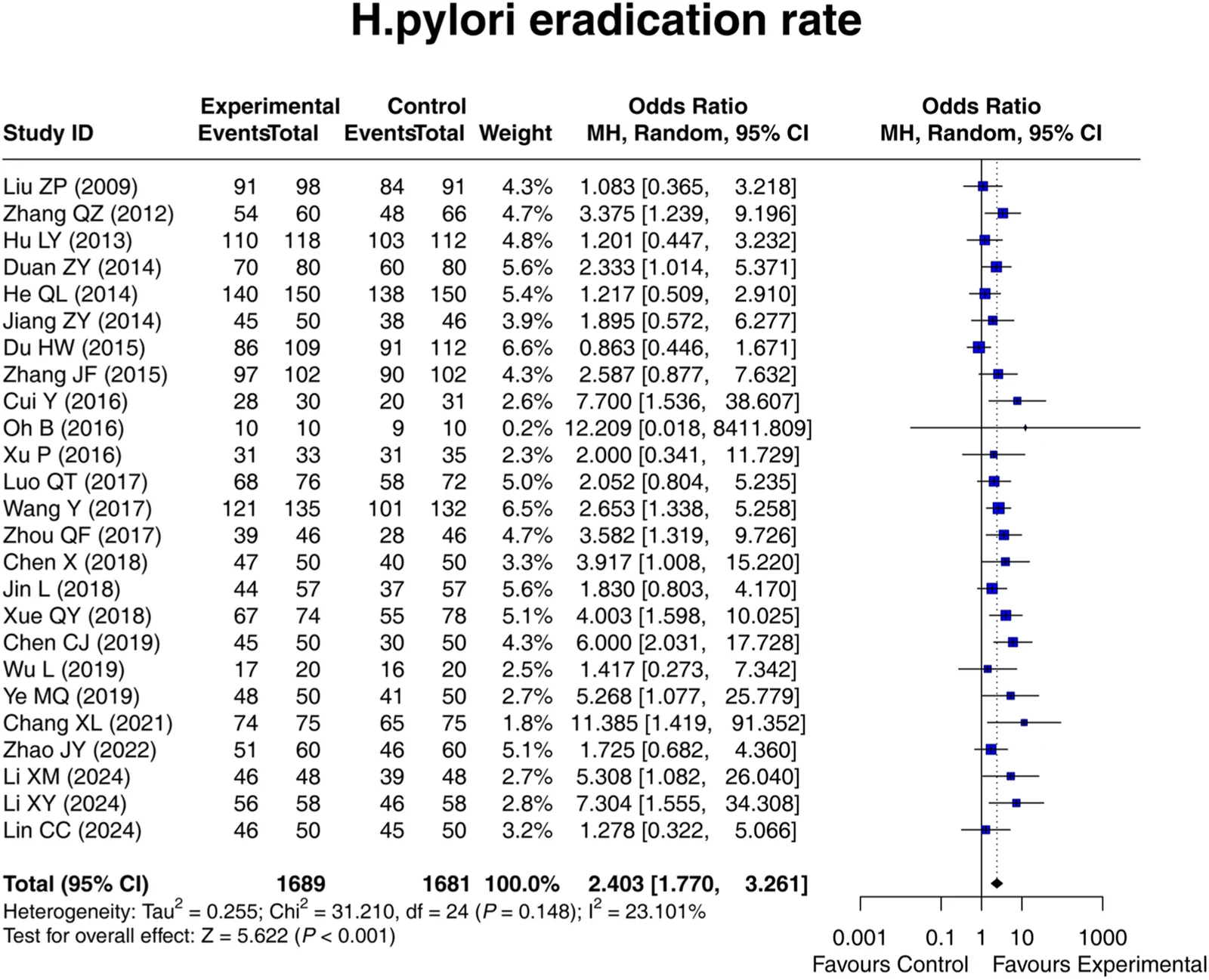
Forest plot of H. pylori eradication rate. H. pylori, Helicobacter pylori.

A total of 25 studies reported the *H. pylori* eradication rate. No heterogeneity existed among studies (*I*^2^ = 23.101%). The random-effects model indicated that the *H. pylori* eradication rate in the experimental group was 2.403 times of that in the control group (OR = 2.403; 95% CI: 1.770, 3.261; p < 0.001). This finding suggested that LCBE-containing interventions enhanced eradication effect compared with interventions without LCBE (Fig. 3).

In the triple therapy subgroup, the *H. pylori* eradication rate in the experimental group was 2.444 times of that in the control group (OR = 2.444; 95% CI: 1.577, 3.788; p < 0.001). In the quadruple therapy subgroup, the *H. pylori* eradication rate in the experimental group was 2.486 times of that in the control group (OR = 2.486; 95% CI: 1.587, 3.894; p < 0.001). Of note, as only one study evaluated LCBE in combination with dual therapy, it was included in the overall analysis but could not be analyzed as a separate subgroup (Supplementary Table 2). In subgroups of LCBE’s daily doses ≤ 1000 mg/day (OR = 2.105; 95% CI: 1.049, 4.226; p = 0.036) and 1500 mg/day (OR = 2.412; 95% CI: 1.678, 3.467; p < 0.001), *H. pylori* eradication rate was increased in experimental group than in the control group. However, in the subgroup of LCBE’s daily doses ≥ 2000 mg/day, *H. pylori* eradication rate was not different between the two groups (OR = 3.106; 95% CI: 0.955, 10.107; p = 0.060) (Supplementary Table 3). In subgroups of treatment durations of ≤ 2 (OR = 2.278; 95% CI: 1.655, 3.135; p < 0.001) and > 2 (OR = 3.938; 95% CI: 1.454, 10.663; p = 0.007) weeks, *H. pylori* eradication rate was increased in experimental group than in the control group (Supplementary Table 4).

### Safety

**Fig. 4:**
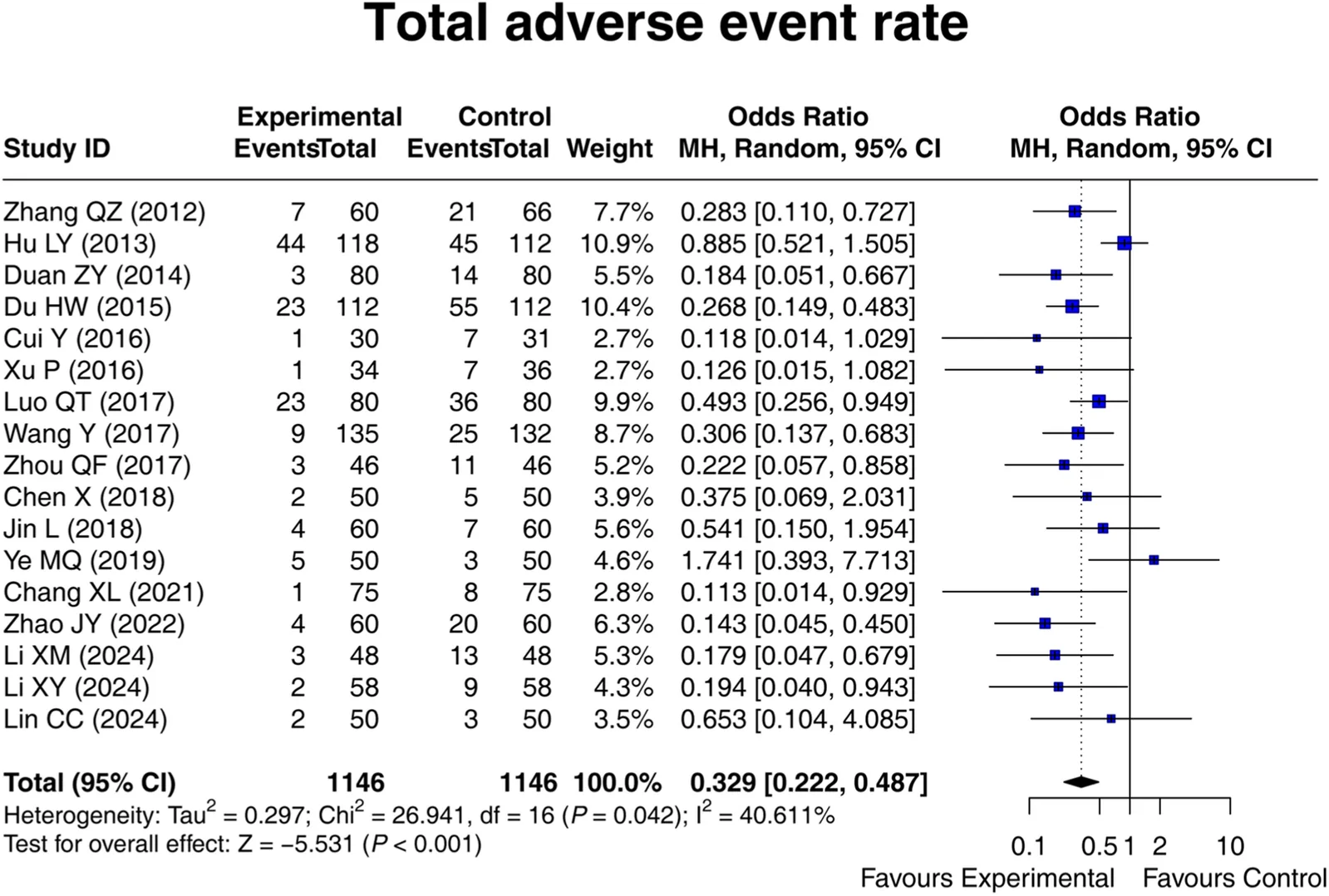
Forest plot of total adverse event rate.

A total of 17 studies reported the total adverse event rate without heterogeneity (*I*^2^ = 40.611%). According to the random-effects model, the total adverse event rate in the experimental group was 0.329-times of that in the control group (OR = 0.329; 95% CI: 0.222, 0.487; p < 0.001), suggesting that LCBE-containing interventions reduced total adverse event compared with interventions without LCBE (Fig. 4).

**Fig. 5:**
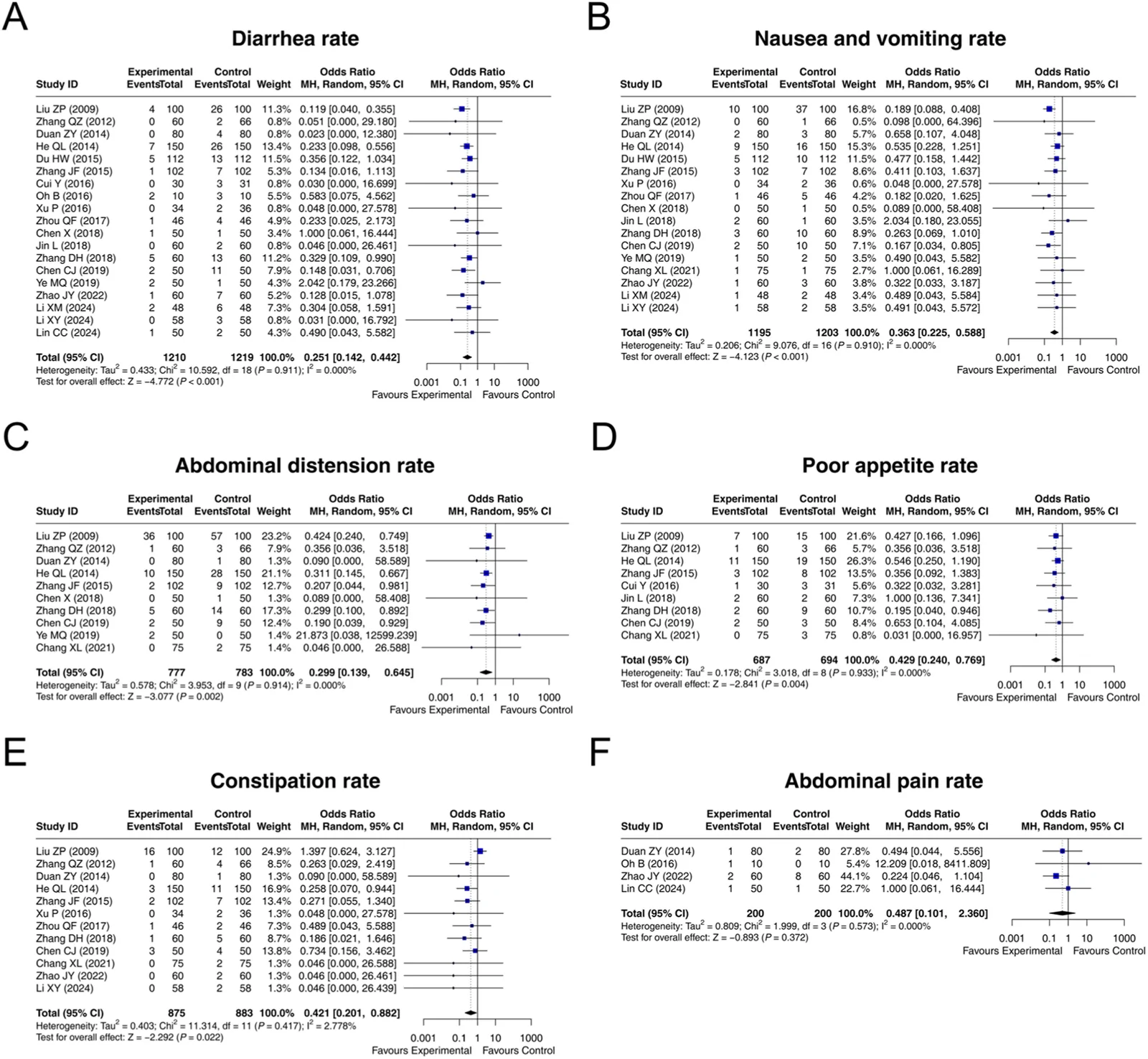
Forest plots of gastrointestinal adverse events. Comparison of the diarrhea rate (A), nausea and vomiting rate (B), abdominal distension rate (C), poor appetite rate (D), constipation rate (E) and abdominal pain rate (F) between the experimental and control groups.

Regarding gastrointestinal adverse events, 19, 17, 10, 9, 12 and 4-studies had data on the diarrhea rate, nausea and vomiting rate, abdominal distension rate, poor appetite rate, constipation rate and abdominal pain rate, respectively. No heterogeneity was observed (all *I*^2^ < 50.000%). The random-effects models revealed that in reference with the control group, the experimental group exhibited 0.251-times of the diarrhea rate (OR = 0.251; 95% CI: 0.142, 0.442; p < 0.001), 0.363 of the nausea and vomiting rate (OR = 0.363; 95% CI: 0.225, 0.588; p < 0.001), 0.299 of the abdominal distension rate (OR = 0.299; 95% CI: 0.139, 0.645; p = 0.002), 0.429 of the poor appetite rate (OR = 0.429; 95% CI: 0.240, 0.769; p = 0.004) and 0.421 of the constipation rate (OR = 0.421; 95% CI: 0.201, 0.882; p = 0.022) of the control group. However, the abdominal pain rate did not differ between the two groups (OR = 0.487; 95% CI: 0.101, 2.360; p = 0.372). The aforementioned findings suggested that LCBE-containing interventions significantly reduced diarrhea, nausea and vomiting, abdominal distension, poor appetite and constipation compared with interventions without LCBE (Fig. 5A‒F).

**Fig. 6:**
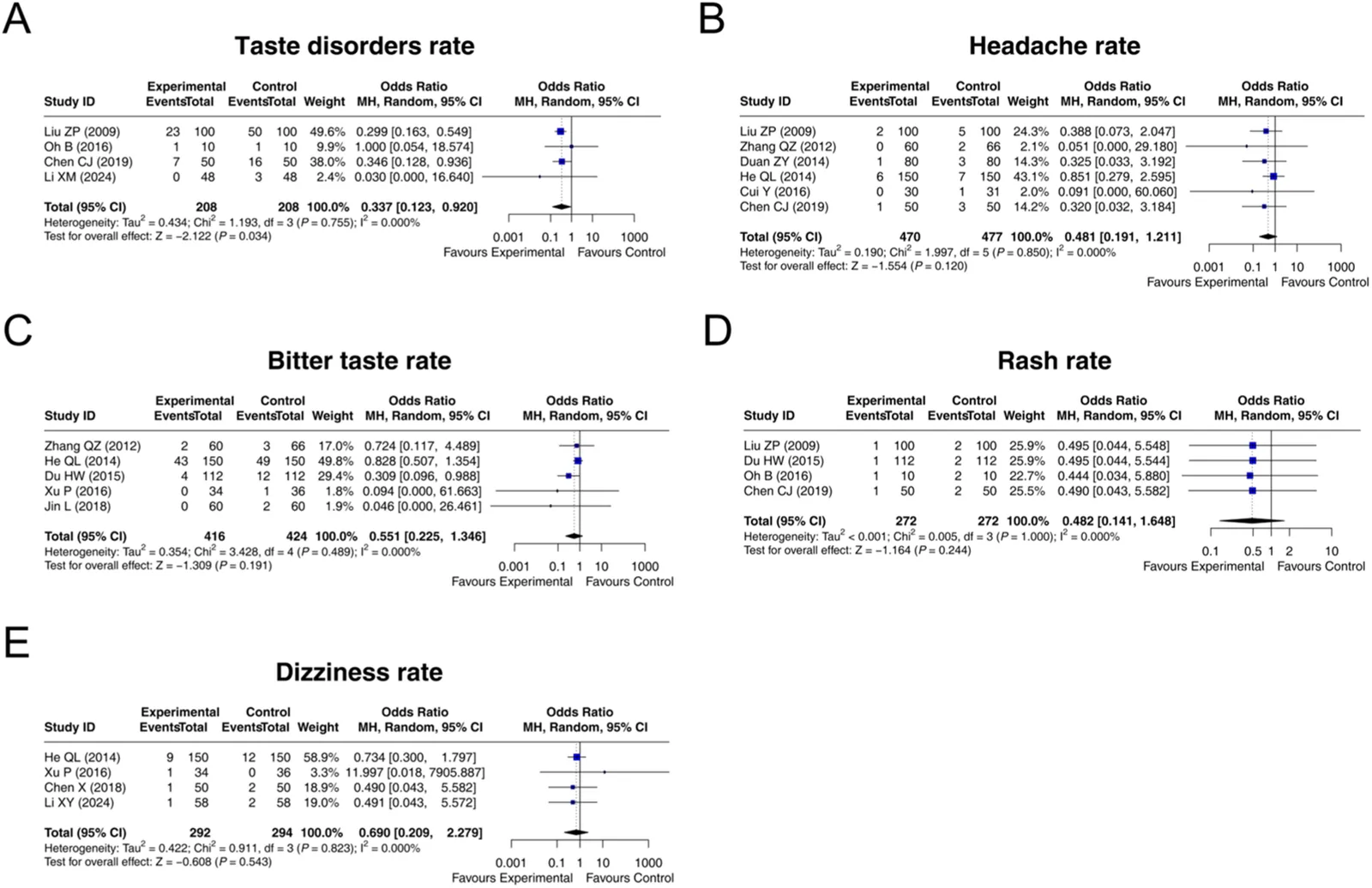
Forest plots of non-gastrointestinal adverse events. Comparison of the taste disorder rate (A), headache rate (B), bitter taste rate (C), rash rate (D) and dizziness rate (E) between the experimental and control groups.

With regard to non-gastrointestinal adverse events, 4, 6, 5, 4 and 4-studies reported taste disorders rate, headache rate, bitter taste rate, rash rate and dizziness rate, respectively, with lack of heterogeneity (all *I*^2^ = 0.000%). According to the random-effects models, the taste disorder rate in the experimental group was 0.337-times of that in the control group (OR = 0.337; 95% CI: 0.123, 0.920; p = 0.034). However, headache rate (OR = 0.481; 95% CI: 0.191, 1.211; p = 0.120), bitter taste rate (OR = 0.551; 95% CI: 0.225, 1.346; p = 0.191), rash rate (OR = 0.482; 95% CI: 0.141, 1.648; p = 0.244), and dizziness rate (OR = 0.690; 95% CI: 0.209, 2.279; p = 0.543) did not differ between the two groups. The aforementioned findings indicated that LCBE-containing interventions significantly decreased the taste disorder compared with interventions without LCBE (Fig. 6A‒E).

### Immunity and inflammation-related parameters

**Fig. 7:**
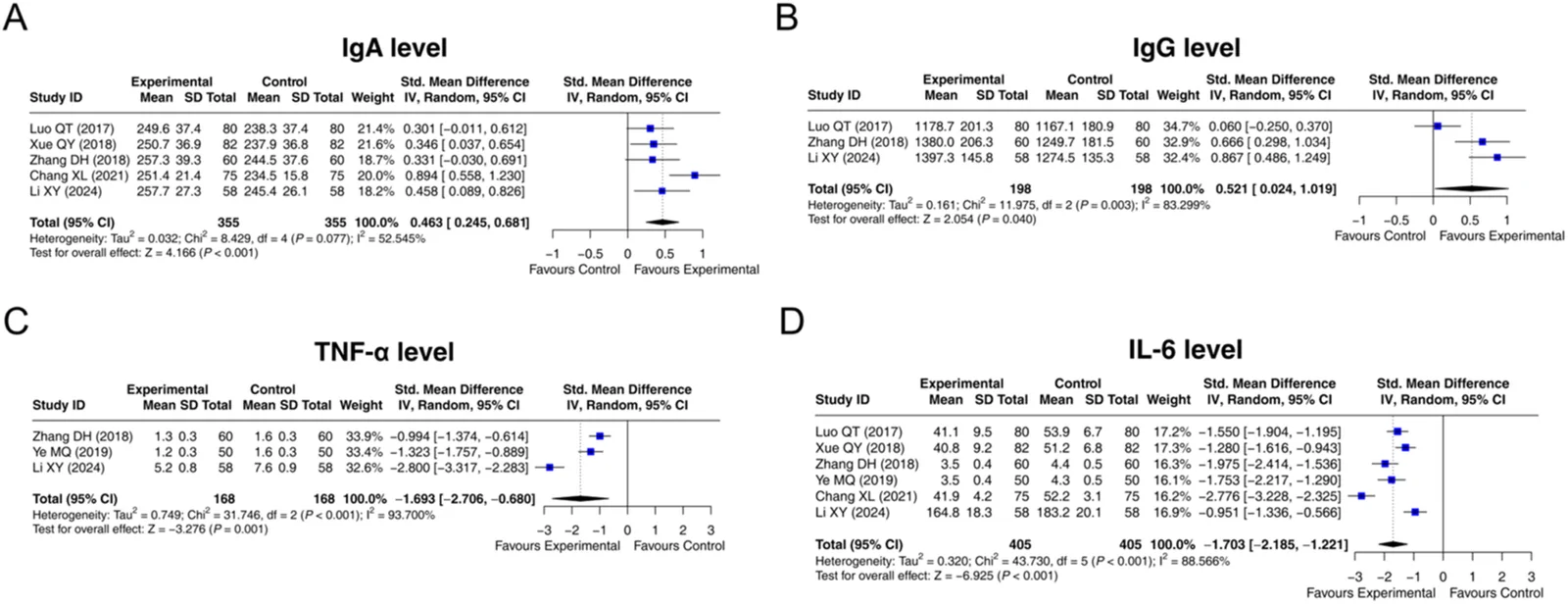
Forest plots of immunity and inflammation-related parameters. Comparison of the IgA (A), IgG (B), TNF-α (C) and IL-6 (D) levels between the experimental and control groups. Ig, Immunoglobulin; TNF-α, Tumor Necrosis Factor-alpha (TNF-α); IL-6, Interleukin-6.

A total of 5, 3, 3 and 6-studies exhibited data on the Immunoglobulin (Ig) A, IgG), Tumor Necrosis Factor-alpha (TNF-α) and Interleukin-6 (IL-6) levels. Heterogeneity was noted in all these outcomes (all *I*^2^ > 50.000%). The random-effects models indicated that compared with the control group, the IgA levels were increased by 0.463 standard deviation units (SMD = 0.463; 95% CI: 0.245, 0.681; p < 0.001); the IgG levels were increased by 0.521 standard deviation units (SMD = 0.521; 95% CI: 0.024, 1.019; p = 0.040), TNF-α levels were decreased by 1.693 Standard Deviation units (SMD = -1.693; 95% CI: -2.706, -0.680; p = 0.001) and IL-6 levels were decreased by 1.703 standard deviation units (SMD = -1.703; 95% CI: -2.185, -1.221; p < 0.001) in the experimental group (Fig. 7A‒D).

### Sensitivity analysis and publication bias

**Fig. 8:**
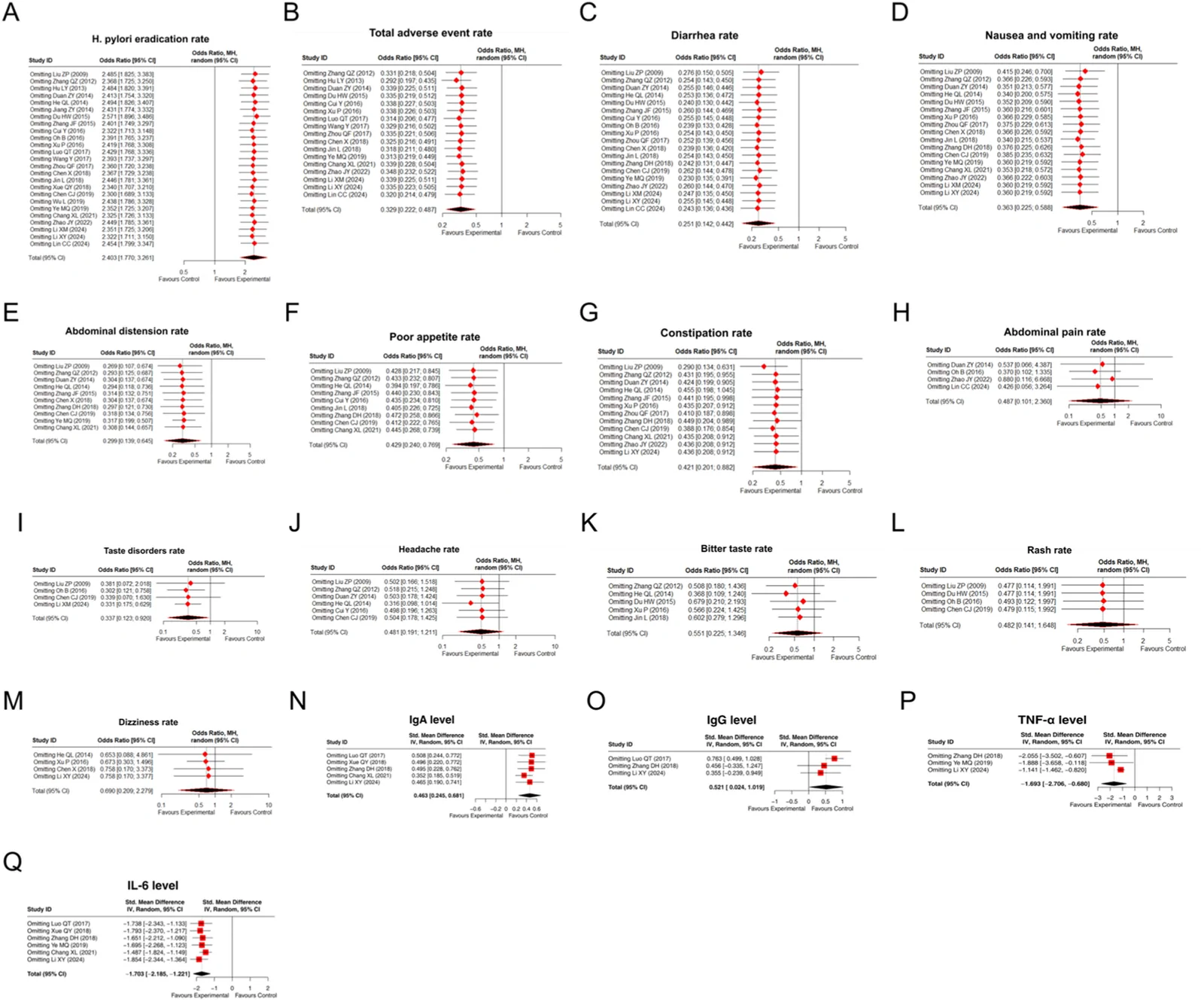
Sensitivity analyses for all outcomes. Sensitivity analyses for H. pylori eradication rate. (A) Total adverse event rate (B), diarrhea rate (C), nausea and vomiting rate (D), abdominal distension rate (E), poor appetite rate (F), constipation rate (G), abdominal pain rate (H), taste disorder rate (I), headache rate (J), bitter taste rate (K), rash rate (L), dizziness rate (M), IgA (N), IgG (O), TNF-α (P), and IL-6 (Q) levels. H. pylori, Helicobacter pylori; Ig, immunoglobulin; TNF-α, Tumor Necrosis Factor-alpha (TNF-α); IL-6, Interleukin-6.

Omitting any of the studies would not alter the outcomes of *H. pylori* eradication rate, total adverse event rate, diarrhea rate, nausea and vomiting rate, abdominal distension rate, poor appetite rate, abdominal pain rate, headache rate, bitter taste rate, rash rate, dizziness rate, IgA levels, TNF-α levels and IL-6 levels. However, the omission of the study of He et al. (2014) would alter the outcome of the constipation rate, the omission of the study of Liu et al. (2009) and Chen et al. (2019) would alter the outcome of the taste disorders rate, the omission of the study by Zhang et al. (2018) and Li et al. (2024) would alter the outcome of the IgG levels (Fig. 8A‒Q).

No publication bias existed regarding the outcomes of *H. pylori* eradication rate, total adverse events rate, diarrhea rate, nausea and vomiting rate, abdominal distension rate, and constipation rate (p > 0.05) (Table 2).

**Table 2:** Publication bias.

**Indicators**	**p-value**	**Bias estimate (SE)**
H. pylori eradication rate	0.070 (Peters’ test)	57.85 (30.42)
Total adverse events rate	0.101 (Peters’ test)	-94.70 (54.10)
Diarrhea rate	0.532 (Peters’ test)	16.83 (26.39)
Nausea and vomiting rate	0.146 (Peters’ test)	-88.16 (57.44)
Abdominal distension rate	0.979 (Peters’ test)	-2.91 (107.73)
Constipation rate	0.326 (Peters’ test)	-128.92 (124.72)

SE, Standard Error.

## Discussion

*H. pylori* infection alters the structure and composition of the gastrointestinal microbiota by regulating the gastrointestinal microecological environment, cytokine production, antimicrobial peptides and immune response, which can further contribute to the development of gastrointestinal diseases.^10^^,^^41^ According to the international consensus, *H. pylori* should be eradicated promptly upon diagnosis.^42^ However, the *H. pylori* eradication rate is below 80% following conventional antibiotic-based therapies.^9^^,^^43^ Fortunately, the addition of probiotics to antibiotic-based therapies could improve *H. pylori* eradication.^44^ As reported by previous meta-analyses, probiotic-containing regimens achieved a higher *H. pylori* eradication than regimens without probiotics.^45^, ^46^, ^47^, ^48^ In line with these previous meta-analyses, the present study demonstrated that LCBE-containing interventions increased the *H. pylori* eradication rate compared with those without LCBE. It was speculated that LCBE may enhance *H. pylori* eradication by producing antimicrobial substances, strengthening the mucosal barrier and competing for adhesion sites.^49^ Subgroup analysis showed that LCBE in combination with triple or quadruple therapy was associated with higher *H. pylori* eradication rate compared with triple or quadruple therapy alone. These findings suggest that LCBE may have potential as an adjunctive component of conventional triple or quadruple therapy. In subgroups of daily doses of ≤ 1000 mg/day and 1500 mg/day, *H. pylori* eradication rate was increased in the experimental group compared to the control group. However, this trend was not found in the subgroup of daily doses of ≥ 2000 mg/day. These findings should be interpreted with caution and do not establish a confirmed dose-response relationship, as the non-significant result in the ≥ 2000 mg/day subgroup may be attributable to the limited number of available studies. Moreover, LCBE-containing interventions were associated with improved eradication rates in both the ≤ 2-week and > 2-week treatment duration subgroups, suggesting that the observed benefit was generally consistent across treatment durations. Overall, these subgroup analyses were exploratory and should be interpreted cautiously; further studies are warranted to validate the potential impact of treatment regimen, LCBE dosage, and treatment duration on the efficacy of LCBE-containing interventions.

Antibiotic-based regimens for *H. pylori* infection often cause gut microbiota dysbiosis, which can lead to the occurrence of adverse events.^3^ Probiotics can alleviate adverse events, such as taste disturbance, diarrhea, nausea and constipation caused by antibiotics in patients with *H. pylori* infection.^10^ In the present meta-analysis, it was shown that the total adverse event rate was reduced by LCBE-containing interventions compared with those without LCBE in patients with *H. pylori* infection. This finding indicated that the addition of LCBE to conventional regimens improved safety profiles compared with conventional regimens alone in patients with *H. pylori* infection. In addition, gastrointestinal adverse events, including diarrhea rate, nausea and vomiting rate, abdominal distension rate, poor appetite rate and constipation rate, were lowered by LCBE-containing interventions compared with those without LCBE in patients with *H. pylori* infection. The potential explanation may be that LCBE could alleviate gut microbiota dysbiosis, thereby reducing gastrointestinal symptoms.^50^^,^^51^ Moreover, the taste disorder rate was also decreased by LCBE-containing interventions compared with those without LCBE. This finding was in line with previous meta-analyses.^52^^,^^53^ However, other non-gastrointestinal adverse events were not significantly different between patients with *H. pylori* infection receiving LCBE-containing interventions and those receiving interventions without LCBE. Therefore, the beneficial effect of LCBE on reducing adverse events may be related to its regulation of gut microbiota.

In *H. pylori* infection, IgA aids the neutralization of pathogens and the maintenance of bacterial communities, while IgG aids in *H. pylori* clearance via immune activation and anti-bacterial activities.^54^ In the present meta-analysis, LCBE-containing interventions increased the IgA and IgG levels in patients with *H. pylori* infection. This finding suggested that LCBE may enhance immune defenses against *H. pylori* infection. Apart from immunity, inflammation also plays a crucial role in *H. pylori* infection.^55^ *H. pylori* infection triggers a cascade of pro-inflammatory responses characterized by upregulated expression levels of cytokines (such as TNF-α and IL-6), which contribute to the pathogenesis of gastrointestinal diseases.^55^ In the present meta-analysis, it was discovered that LCBE-containing interventions reduced TNF-α and IL-6 levels in patients with *H. pylori* infection. As explained by a previous study, LCBE may attenuate intestinal inflammation by enhancing fecal microbiome metabolism and the colonic barrier.^12^

The present meta-analysis contained several limitations. 1) It was disclosed that compared with conventional treatments, LCBE-containing interventions exhibited improved efficacy and safety in patients with *H. pylori* infection. However, it was unclear whether LCBE was superior to other probiotics in treating *H. pylori* infection. 2) The exploration of medication adherence was crucial for optimizing therapeutic outcomes. However, insufficient data from available studies hindered further exploration of this area. Additional studies should be conducted to compare the adherence between patients receiving intervention with or without LCBE. 3) LCBE-containing interventions increased IgA and IgG levels. However, the underlying mechanisms deserved to be investigated further. 4) The predominance of studies was conducted in China, which might limit the generalizability of our findings to other populations.

In summary, LCBE-containing interventions are associated with higher eradication rates, fewer adverse events, and favorable changes in immune and inflammatory markers in patients with *H. pylori* infection.

## Authors’ contributions

Chenghai Yang: Conceptualization; formal analysis; methodology; project administration; writing-original draft; writing-review & editing.

Bingyun Lu: Data curation; investigation; resources; writing-original draft; writing-review & editing.

Cangui Zhang: Data curation; investigation; resources; writing-original draft; writing-review & editing.

Anjiang Wang: Data curation; investigation; resources; writing-original draft; writing-review & editing.

Ye Chen: Conceptualization; Formal analysis; methodology; supervision; writing-original draft; writing-review & editing.

## Ethics approval

Not applicable.

### Informed consent statement

Not applicable.

## Funding

This study was supported by the 10.13039/501100001809National Natural Science Foundation of China (No. 82270581) and 10.13039/501100012235Baoan District Medical and Health Scientific Research Project of Shenzhen City (No. 2023JD240).

## Conflicts of interest

The authors declare no conflicts of interest.

## Data Availability

The data must be requested from the corresponding author.
