# Supplementary material for: Efficacy and safety of live combined Bacillus subtilis and Enterococcus faecium in patients with Helicobacter pylori infection: A meta-analysis of randomized controlled trials
Source: Clinics (Sao Paulo). 2026 Sep 10;81:101139. doi: 10.1016/j.clinsp.2026.101139 (PMC13586577; doi:10.1016/j.clinsp.2026.101139)
Supplement: Supplementary file 1 [file mmc1.docx]

CLINICS-D-25-02209_Supplementary Material

**Supplementary Table 1** Detailed search strategy for PubMed and Web of Science database.

| **Database** | **Search strategy** |
| --- | --- |
| PubMed | ((Bacillus Subtilis AND Enterococcus Faecium) OR (Live Combined Bacillus Subtilis and Enterococcus Faecium) OR (Probiotics)) AND ((Helicobacter pylori eradication) OR (*H. pylori* eradication)) |
| Web of Science | ((((TS=(Bacillus Subtilis AND Enterococcus Faecium)) OR TS=(Live Combined Bacillus Subtilis and Enterococcus Faecium)) OR TS=(Probiotics)) AND TS=(Helicobacter pylori eradication)) AND TS=(*H. pylori* eradication) |
| Cochrane Library | #1, Bacillus Subtilis AND Enterococcus Faecium  #2, Live Combined Bacillus Subtilis and Enterococcus Faecium  #3, Probiotics  #4, #1 OR #2 OR #3  #5, Helicobacter pylori eradication  #6, H. pylori eradication  #7, #5 OR #6  #8, #4 AND #7 |
| CNKI | 主题“枯草杆菌二联活菌 + 益生菌 + 微生物制剂” AND 主题“幽门螺杆菌 + 幽门螺旋杆菌” |
| Wan Fang | (枯草杆菌二联活菌 OR 益生菌 OR 微生物制剂) AND (幽门螺杆菌 OR 幽门螺旋杆菌) AND (感染 OR 根治） |
| VIP | 题名或关键字“枯草杆菌二联活菌 OR 益生菌 OR 微生物制剂” AND 题名或关键字“幽门螺杆菌+helicobacter pylori+helicobacter pylori+helicobacter pylon+helicobacter pylori+helicobactor pylori+幽门螺旋菌+幽门螺旋杆菌+螺旋菌” |

**Supplementary Table 2** Subgroup analysis of *H. pylori* eradication rate in different drug combination regimens.

| **Items** | **Triple therapy** | **Quadruple therapy** |
| --- | --- | --- |
| Number of studies | 12 | 12 |
| Heterogeneity test |  |  |
| *I*^2^ | 3.860% | 41.583% |
| Model selected | Random effect model | Random effect model |
| Pooled odds ratio [95% CI] | 2.444 [1.577, 3.788] | 2.486 [1.587, 3.894] |
| Test for overall effect |  |  |
| Z | 3.997 | 3.978 |
| p-value | <0.001 | <0.001 |

CI, Confidence Interval.

**Supplementary Table 3** Subgroup analysis of *H. pylori* eradication rate in different total daily doses.

| **Items** | **≤1000 mg/day** | **1500 mg/day** | **≥2000 mg/day** |
| --- | --- | --- | --- |
| Number of studies | 5 | 18 | 2 |
| Heterogeneity test |  |  |  |
| *I*^2^ | 7.413% | 27.009% | 65.938% |
| Model selected | Random effect model | Random effect model | Random effect model |
| Pooled odds ratio [95% CI] | 2.105 [1.049, 4.226] | 2.412 [1.678, 3.467] | 3.106 [0.955, 10.107] |
| Test for overall effect |  |  |  |
| Z | 2.093 | 4.759 | 1.883 |
| p-value | 0.036 | <0.001 | 0.060 |

CI, confidence interval.

**Supplementary Table 4** Subgroup analysis of *H. pylori* eradication rate in different treatment durations.

| **Items** | **≤2 weeks** | **>2 weeks** |
| --- | --- | --- |
| Number of studies | 22 | 3 |
| Heterogeneity test |  |  |
| *I*^2^ | 20.263% | 7.858% |
| Model selected | Random effect model | Random effect model |
| Pooled odds ratio [95% CI] | 2.278 [1.655, 3.135] | 3.938 [1.454, 10.663] |
| Test for overall effect |  |  |
| *Z* | 5.051 | 2.697 |
| p-value | <0.001 | 0.007 |

CI, Confidence Interval.

**Supplementary Figure 1** Risk of bias in each study.
